# Reproductive ecology of interior least tern and piping plover in relation to Platte River hydrology and sandbar dynamics

**DOI:** 10.1002/ece3.2964

**Published:** 2017-04-10

**Authors:** Jason M. Farnsworth, David M. Baasch, Chadwin B. Smith, Kevin L. Werbylo

**Affiliations:** ^1^ Platte River Recovery Implementation ProgramKearneyNEUSA

**Keywords:** central Platte River, hydrology, interior least tern, lower Platte River, piping plover, reproductive success, sandbar height distributions, stage–discharge relationships

## Abstract

Investigations of breeding ecology of interior least tern (*Sternula antillarum athalassos*) and piping plover (*Charadrius melodus*) in the Platte River basin in Nebraska, USA, have embraced the idea that these species are physiologically adapted to begin nesting concurrent with the cessation of spring floods. Low use and productivity on contemporary Platte River sandbars have been attributed to anthropomorphically driven changes in basin hydrology and channel morphology or to unusually late annual runoff events. We examined distributions of least tern and piping plover nest initiation dates in relation to the hydrology of the historical central Platte River (CPR) and contemporary CPR and lower Platte River (LPR). We also developed an emergent sandbar habitat model to evaluate the potential for reproductive success given observed hydrology, stage–discharge relationships, and sandbar height distributions. We found the timing of the late‐spring rise to be spatially and temporally consistent, typically occurring in mid‐June. However, piping plover nest initiation peaks in May and least tern nest initiation peaks in early June; both of which occur before the late spring rise. In neither case does there appear to be an adaptation to begin nesting concurrent with the cessation of spring floods. As a consequence, there are many years when no successful reproduction is possible because emergent sandbar habitat is inundated after most nests have been initiated, and there is little potential for successful renesting. The frequency of nest inundation, in turn, severely limits the potential for maintenance of stable species subpopulations on Platte River sandbars. Why then did these species expand into and persist in a basin where the hydrology is not ideally suited to their reproductive ecology? We hypothesize the availability and use of alternative off‐channel nesting habitats, like sandpits, may allow for the maintenance of stable species subpopulations in the Platte River basin.

## 1 Introduction

**Figure 1:**
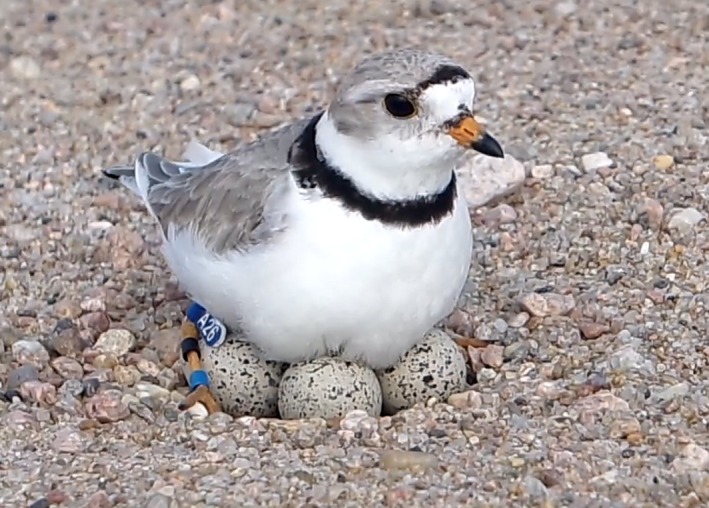
Piping plover tending its nest

Interior least tern (*Sternula antillarum athalassos*; hereafter, least tern) and piping plover (*Charadrius melodus*) (Figure 1) are two species of endangered and threatened birds that nest on barren to sparsely vegetated riverine sandbars, sand and gravel pits, and along lake shorelines in North America (USFWS, 1990). The Platte River Recovery Implementation Program (Program) has been tasked with improving least tern and piping plover use and productivity along 145 km of the big bend reach of the Platte River in central Nebraska, USA (NAD83, zone 14, UTM‐X—504100; UTM‐Y—4501000). Program activities in this reach, known as the Associated Habitat Reach (AHR), are intended to mitigate declines in species habitat suitability due to water development in the Platte River basin (Department of the Interior, 2006). The decline in AHR habitat suitability has been inferred from (1) the body of evidence documenting a substantial change in central Platte River (CPR) hydrology and associated reduction in unvegetated channel width over historical timeframes, (2) the presence of species nesting on off‐channel habitat, but lack of suitable sandbar nesting habitat and on‐channel productivity in the contemporary CPR, and (3) species use of riverine habitat in the contemporary lower Platte River (LPR) which experiences higher peak flow magnitudes. Implicit in this inference are the assumptions that on‐channel productivity in the LPR is sufficient to maintain stable subpopulations and the LPR is an analog for the historical CPR prior to water development.

The first investigation of breeding ecology of least tern and piping plover along the CPR was conducted in 1979 (Faanes, 1983). Faanes located 17 least tern and 40 piping plover nests on river sandbars. All nests were inundated by rising water on 21 June at a discharge of 3,000 cfs. Faanes concluded the 1979 late spring discharge was highly altered because of late Rocky Mountain snowmelt and heavy rainfall and cited Hardy's (1957) suggestion of a relationship between nesting and cessation of spring floods. Subsequent investigations of breeding ecology of least tern and piping plover in the Platte River basin have embraced this concept, stating these species are adapted to begin nesting in the CPR after water levels recede and sandbars are exposed in the spring (Department of the Interior 2006; Kirsch, 1996; Sidle, Dinan, Dryer, Rumancik, & Smith, 1988).

The hydrology of the CPR and LPR is characterized by two spring rises, one in early spring due to localized snowmelt and one in the late spring due to snowmelt and precipitation runoff from basin headwaters in the high plains and Rocky Mountains in Colorado and Wyoming, USA (Murphy, Randle, Fotherby, & Daraio, 2004). If, as hypothesized, least tern and piping plover are physiologically adapted to begin nesting on the Platte River concurrent with the recession of the spring rise, we would expect this to be reflected in the timing of species nest initiation. This adaptation is apparent in analyses of least tern nesting data on the lower Mississippi River where the annual hydrograph peaks in April and least tern nest initiation period begins in May, following the peak (Dugger, Ryan, Galat, Renken, & Smith, 2002). Within the contemporary AHR and LPR, however, piping plovers nest from late April to early August with the highest proportion of nests being initiated during May. Least terns breed and nest from mid‐May to early August with the highest nesting incidence occurring in early June. As a result, a majority of nests are often initiated prior to the late spring rise and are susceptible to loss from inundation.

The relationship between hydrology, sandbar habitat, and species ecology has been explored in other river systems (Catlin et al., 2010; Dugger et al., 2002; Jorgensen, 2009). However, there have been few attempts to quantitatively evaluate differences through comparative analyses. In this investigation, we endeavored to (1) examine the timing of the late spring rise in relation to least tern and piping plover nesting ecology on the historical and contemporary CPR and the contemporary LPR and (2) compare and contrast the potential for on‐channel species productivity in the CPR and LPR segments given our current understanding of basin hydrology, channel hydraulics, and sandbar height relationships.

## 2 Methods

### 2.1 Study area

**Figure 2:**
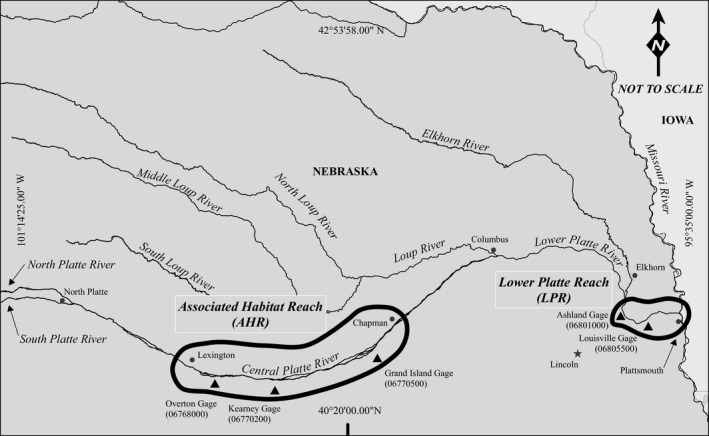
Location of Associated Habitat Reach (AHR) and lower Platte River (LPR) study reaches and stream gages

We included two segments of the Platte River in Nebraska in our study (Figure 2). The AHR in central Nebraska, USA, is a 145‐km stretch of river extending from Lexington downstream to Chapman, Nebraska. The LPR study area is a 53‐km stretch of river extending from the confluence of the Elkhorn River to the Missouri River near Plattsmouth, Nebraska. This segment has the highest incidence of on‐channel nesting in the Platte River basin.

### 2.2 Species nest initiation in relation to Platte River hydrology

We computed the mean annual hydrograph for the historical AHR and contemporary AHR and LPR reaches from mean daily discharge records and plotted them against the distribution of AHR least tern and piping plover nest initiation dates to evaluate the relative timing of species nest initiations periods in relation to annual peaks. A more detailed within‐year analysis of nesting in relation to peak flows was not possible due to the lack of systematically collected season‐long monitoring data in the historical AHR and contemporary LPR reaches.

#### 2.2.1 Nest and brood exposure data

We compiled the specific dates least tern and piping plover initiate nests, hereafter referred to as nest initiation dates, from all on‐ and off‐channel CPR monitoring data for the period of 2001–2013 (Baasch, 2014) and used standard Program nest exposure periods (nest initiation to chick fledging) to establish the nesting and brood rearing period for each species (Baasch, Hefley, & Cahis, 2015). To eliminate the disproportionate effect of early and late nests on the length of the nest initiation season, we used the 5th and 95th percentile of the nest initiation dates to define the nest initiation window. A quantitative analysis of on‐channel nest initiation dates in relation to peak discharge dates was not possible given the paucity of on‐channel nesting in the CPR and lack of season‐long systematic monitoring data for the LPR.

#### 2.2.2 Annual hydrograph

Mean daily flow observations in the historical AHR (1895–1938) were of specific interest in this study. However, with the exception of a 5‐year period from 1902 to 1906, they were unavailable prior to 1915 (Stroup, Rodney, & Anderson, 2006). Mean daily flows were, however, available upstream on the North Platte River near North Platte, Nebraska in all years except 1910 and on the North Platte River above Lake McConaughy in all years except 1913–1914 (Stroup et al., 2006). We used a flow record extension technique, Maintenance of Variance Extension Type 1 (MOVE.1; Hirsch, 1982), to estimate mean daily flows on the Platte River near Overton, Nebraska from 1895 to 1914 using upstream flow observations. We assessed model performance by comparing MOVE.1 estimated and observed Platte River flows near Overton, Nebraska, 1902–1906 using Nash Sutcliffe Coefficient of Efficiency (NSCE; Nash & Sutcliffe, 1970). NSCE values exceeded 0.70 which was deemed satisfactory and, as summarized by Moriasi et al. (2007), are in the general range of reported NSCE values when modeling flow. We combined the observed and estimated daily discharge records (1895–1914) with records from USGS Gage 06768000 at Overton (1915–1938) to produce a 44‐year historical AHR data series.

We retrieved daily discharge records for the contemporary CPR and LPR reaches from the U.S. Geological Survey (USGS, 2001) National Water Information System (NWIS) for the period of 1954–2012, which was the longest concurrent period of record for both the CPR and LPR gages. We used gage 06770500 at Grand Island, Nebraska for AHR hydrology and gage 06805500 at Louisville, Nebraska for LPR hydrology.

### 2.3 Emergent sandbar availability model

We developed a simple deterministic model to estimate the annual availability of emergent sandbar habitat during the nesting season using discharge records, stage‐discharge relationships, and observed sandbar heights. Model input and output variables are listed in Table 1.

**Table 1:** Input and output variables for the emergent sandbar habitat model

Model input variables	
DISCH_HAB_	Maximum of mean daily flow (cm) from 1 January of the previous year through 1 July of analysis year. Considered to be the discharge that controlled sandbar height in analysis year
STAGE_HAB_	River stage (m) associated with DISCH_HAB_
BAR HEIGHT	Sandbar height (m) below peak stage.
STAGE_BAR_	Stage (m) of sandbars
DISCH_DAILY_	Daily river discharge (cm)
STAGE_DAILY_	Daily river stage (m)
Model output variables	
SUCCESS WINDOW_PLOVER_	Number of days when piping plover nests could be initiated, incubated, and hatch and the chicks successfully fledged without being inundated.
SUCCESS WINDOW_TERN_	Number of days when least tern nests could be initiated, incubated, and hatch and the chicks successfully fledged without inundation.

Model operations/calculations for each analysis year included:

1. Identify maximum daily discharge for the period from 1 January the year prior to each analysis year and ending 1 July of the analysis year (hydrology methods presented in Section 2.2.2). We considered maximum flow during this period to be the habitat‐forming discharge (DISCH_HAB_) controlling the height of sandbars in the analysis year. The 1.5‐year period for identification of DISCH_HAB_ allowed for sandbar persistence through two nesting seasons.
2. Calculate stage (STAGE_HAB_) of the habitat‐forming discharge for each year using DISCH_HAB_ and gage stage–discharge relationship (stage–discharge relationships presented in Section 2.3.1).
3. Calculate the stage associated with sandbars (STAGE_BAR_) for each nesting season by subtracting sandbar height (BAR HEIGHT) relative to peak stage (see Section 2.3.2 for sandbar height relationships) from STAGE_HAB_.
4. Calculate daily stage (STAGE_DAILY_) during the least tern and piping plover nesting and brood rearing seasons of each year using mean daily discharge and stage–discharge relationships.
5. Compare daily river stage (STAGE_DAILY_) to sandbar stage (STAGE_BAR_) to determine whether bar height exceeded river stage (i.e., were emergent).
6. Calculate the maximum number of contiguous days during each nesting and brood rearing seasons (Section 2.4) when bars were emergent.
7. Subtract period for successful nesting and brood rearing (64 days for piping plovers and 49 for least terns; Table 2) from maximum contiguous days with emergent sandbars to determine the number of days during each nesting season when a nest could have been initiated and successfully fledge chicks without being inundated (success window).

**Table 2:** Ninetieth percentile of least tern and piping plover nesting and brood rearing dates within the Associated Habitat Reach (AHR), 2001–2013

Nest exposure metric	Piping plover	Interior least tern
Nest count (number of nests)	287	770
Nest initiation and egg laying period (days)^a^	8	3
Incubation period (days)	28	21
Brooding period (days)	28	21
Period for successful nesting (days)^b^	64	45
First nest initiation date (day‐month)	1‐May	28‐May
First hatch date (day‐month)^c^	6‐June	21‐June
First fledge date (day‐month)^d^	4‐July	12‐July
Median nest initiation date (day‐month)	15‐May	10‐June
Median hatch date (day‐month)	20‐June	8‐July
Median fledge date (day‐month)	18‐July	29‐July
Last nest initiation date (day‐month)	23‐June	16‐July
Last hatch date (day‐month)	29‐July	9‐August
Last fledge date (day‐month)	26‐August	30‐August
Nesting initiation window (days)	118	95

a Nest initiation date was determined by the date a nest (scrape with ≥1 egg) was first observed or by egg floating techniques.

b Nest initiation and egg‐laying period + incubation period + brooding period.

c Hatch date was determined by observations of ≥1 chick or was estimated based on chick age.

d Fledge date was determined by the earlier date between first observing sustained flight and a predefined fledging age for each species.

#### 2.3.1 Hydraulics (stage–discharge relationships)

We used stream gage stage–discharge rating curves to characterize river hydraulics in the contemporary reaches in an effort to be consistent with previous analyses (Jorgensen, 2009; Parham, 2007). Critiques of similar analyses in other systems cautioned that use of hydraulic data at gage locations may not be representative of the geomorphic variability of a river system, specifically in reaches with least tern and piping plover nesting (Catlin et al., 2010; Jorgensen, 2009). To address this concern, we compared stage–discharge relationships at gage locations to best‐available hydraulic data at nest sites.

**Figure 3:**
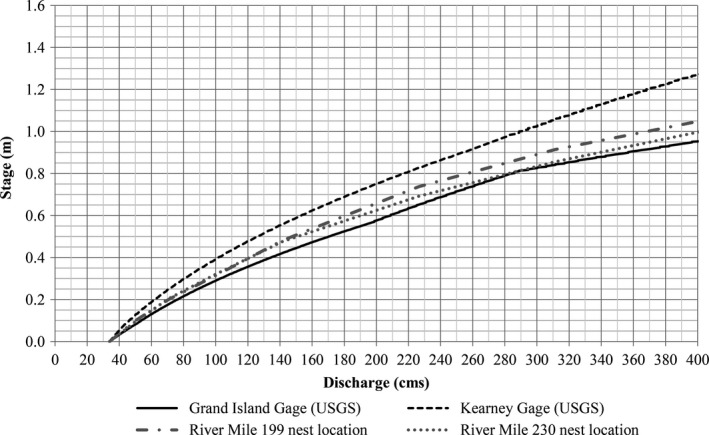
Comparison of contemporary Grand Island (06770500) and Kearney (06770200) stream gage stage–discharge relationships and HEC‐RAS model stage–discharge relationships at river kilometer 515 and 595 in the AHR. All relationships were normalized to a stage of 0.0 m at 34 cm for comparison. The stage–discharge relationship at the Grand Island gage was within 0.09 m of the relationships at the nest locations throughout the discharge range and the shape of the curves was very similar

In the contemporary AHR, limited nesting has occurred on sandbars at river kilometers 320 and 370 (Baasch, 2014). We compared modeled HEC‐RAS stage–discharge relationships (HDR Inc. et al., 2011) at these locations to USGS stage–discharge rating curves for the Kearney and Grand Island, Nebraska gages and determined the Grand Island gage relationship was the most representative of nesting colony locations within the AHR (Figure 3).

**Figure 4:**
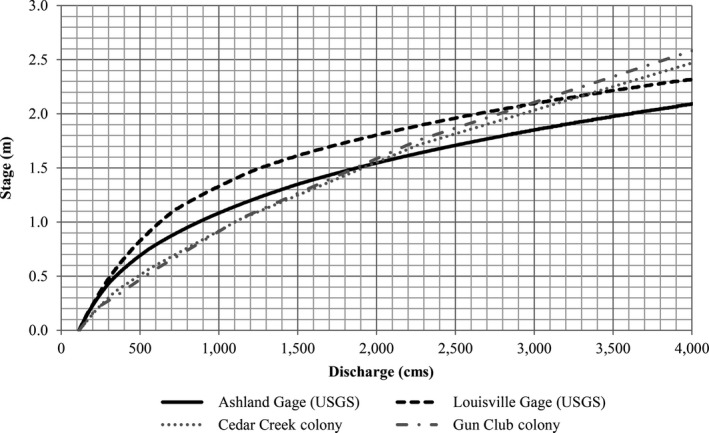
Comparison of Louisville (06805500) and Ashland (06801000) stream gage stage–discharge relationships and FEMA HEC‐2 model stage–discharge relationships at Cedar Creek and Gun Club colony locations in the lower Platte River (LPR). All relationships were normalized to a stage of 0.0 m at 113 cm for comparison

In the LPR, we compared USGS stage–discharge relationships at the Louisville and Ashland, Nebraska gages to Federal Emergency Management Agency HEC‐2 hydraulic model (HDR Inc. et al., 2009) stage–discharge relationships in the Cedar Creek and Gun Club reaches which have consistently supported nesting (Brown & Jorgensen, 2008, 2009, 2010; Brown, Jorgensen, & Dinan, 2011, 2012, 2013) and determined the Ashland gage to be the most representative (Figure 4).

No stream gage stage–discharge relationships exist for the historical AHR. As such, we used a stage–discharge relationship from a HEC‐RAS hydraulic model of the historical channel near Odessa, Nebraska (Simons & Associates Inc., 2012). It was not possible to directly assess the representativeness of the stage–discharge relationship for the historical AHR. However, we compared channel width in the modeled reach near Odessa, Nebraska (1,300 m) to that of the channel near Lexington, Nebraska, (1,220 m) where the earliest on‐channel nesting in the AHR was observed (Wycoff, 1960). The similarity of width provides some confidence the modeled stage–discharge relationship is reasonable.

**Figure 5:**
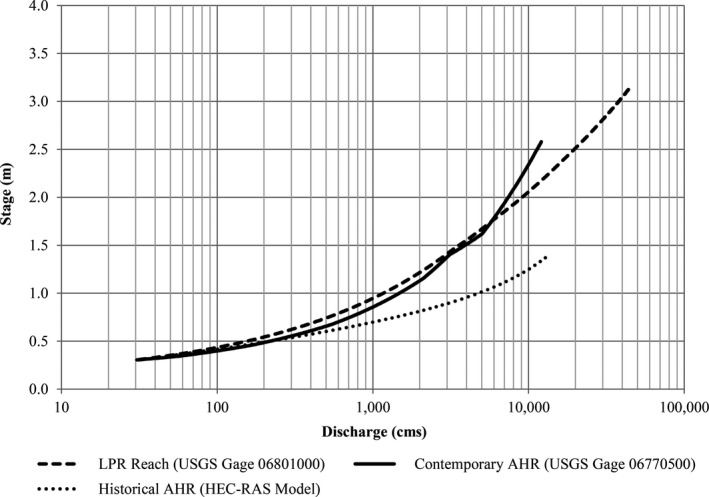
Stage–discharge relationships used for model reaches. All relationships normalized to a stage of 0.3 m at 30 cm for comparison

**Figure 6:**
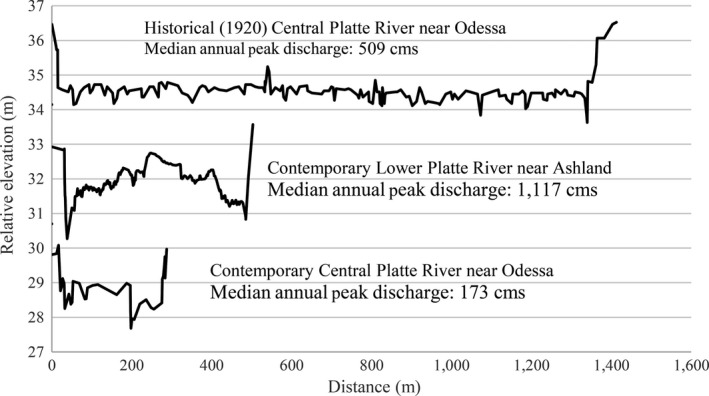
Channel width and median annual peak discharge comparison for model reaches. Note, the historical Associated Habitat Reach (AHR) was substantially wider than the contemporary lower Platte River (LPR) Reach and median annual peak flow was 55% lower

The stage–discharge relationships for the contemporary AHR and LPR Reaches are similar (Figure 5). However, the stage increase with discharge in the historical AHR was somewhat lower than the contemporary LPR reach. The reason for this disparity is apparent from a channel cross section comparison. The historical AHR was much wider than the contemporary LPR reach despite having somewhat lower mean annual and median annual peak discharges (Figure 6).

#### 2.3.2 Sandbar heights

**Figure 7:**
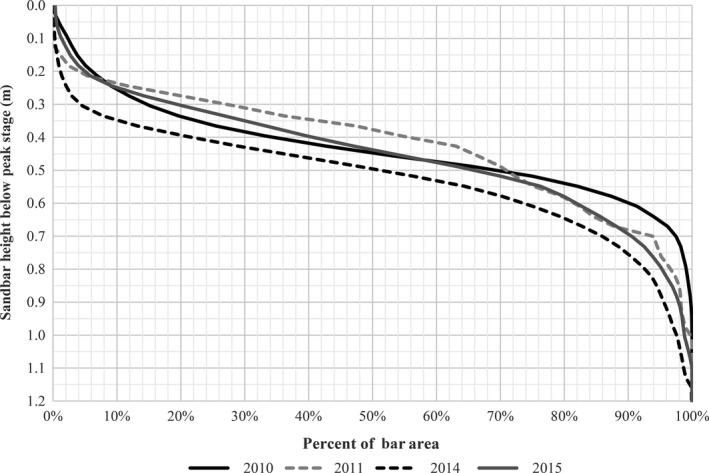
Cumulative distributions of Associated Habitat Reach (AHR) sandbar heights following sandbar forming peak flow events in 2010 (226 cm), 2011 (255 cm), 2014 (198 cm), and 2015 (425 cm)

We used a combination of remote‐sensing data and hydraulic modeling data to estimate distributions of sandbar heights relative to peak stage in the contemporary AHR following natural high‐flow events that occurred in 2010, 2011, 2014, and 2015. Event peak magnitudes ranged from 190 to 434 cm and event durations ranged from 33 to 98 days. The median sandbar height in the AHR across all years was 0.46 m below peak stage (Program unpublished report; Figure 7). The USGS conducted field surveys of sandbar topography in the LPR following the 2010 high‐flow event and generated a similar sandbar height distribution (Alexander, Schultze, & Zelt, 2013). The median height in the LPR following the 2010 event was 0.61 m below peak stage (Alexander et al., 2013). A sandbar height potential of 0.46 m below peak stage was used for the contemporary AHR model and 0.61 m was used for the LPR model.

Median bed material grain size in the contemporary AHR is approximately 0.96 mm and in the LPR is 0.22 mm. The slightly lower sandbar heights relative to peak stage observed in the LPR are consistent with published bedform height relationships in which height decreases as bed material grain size decreases (Ikeda, 1984; Julien & Klaassen, 1995; Van Rijn, 1984). The median bed material grain size of the historical AHR of approximately 0.40 mm (USACE, 1931) was finer than the contemporary AHR (0.96 mm) and coarser than the LPR (0.22 mm). Consequently, median sandbar height potential in the historical AHR would be expected to be lower than the contemporary AHR and higher than the contemporary LPR. We elected to use the contemporary AHR median sandbar height of 0.46 m to provide a conservatively high estimate of sandbar heights in the historical AHR.

### 2.4 Emergent sandbar availability model performance and sensitivity

We qualitatively assessed the performance of the model through comparison of model results with recorded observations of nest loss due to inundation, focusing on discharges that inundated nests in relation to habitat forming discharge. We assessed the sensitivity of success window to stage–discharge relationships and sandbar heights using Oracle^®^ Crystal Ball software. We ran Monte Carlo simulations with triangular distributions of stage per unit discharge ranging from 70% to 130% of the USGS rating curves, approximating the range of observed stage–discharge relationships in both reaches. We also varied sandbar heights by ±0.46 m from the observed mean value to represent bar height potential ranging from peak stage to approximately 1 m below peak stage. Each input variable's contribution to variance in species success window output was used to assess sensitivity.

## 3 Results

### 3.1 Species nest initiation in relation to the annual hydrograph of the Platte River

The contemporary AHR nest initiation window for piping plovers was 1 May–23 June and was 28 May–16 July for least terns (Table 2). Approximately 90% of on‐channel least tern and piping plover nest initiation dates reported on the LPR during the period of 2008–2013 also fell within the same timeframes (Brown & Jorgensen, 2008, 2009, 2010; Brown et al., 2011, 2012, 2013). The entire nesting and brood rearing season for piping plovers encompassed the period from 1 May–26 August and 28 May–30 August for least terns (Table 2).

**Figure 8:**
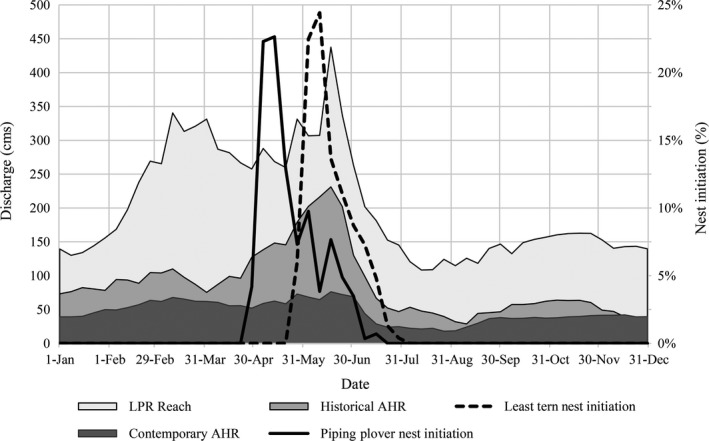
Distribution of Associated Habitat Reach (AHR) piping plover nest initiation dates (2001–2013) in relation to the annual hydrographs of the lower Platte River (LPR) (1954–2012), contemporary AHR (1954–2012), and historical AHR (1895–1938)

Two spring rises are evident in the annual hydrographs of the historical AHR, contemporary AHR, and contemporary LPR (Figure 8). The first occurs in the February–March period and the second peak occurs in mid‐June. The peaks are less defined in the contemporary AHR due to the flow damping influence of storage reservoirs (Simons & Associates Inc. and URS Greiner Woodward Clyde, 2000). The beginning of the piping plover nest initiation window coincides with the end of the early spring rise, but peaks a month prior to the late‐spring rise in June (Figure 3). Consequently, the late‐spring rise often occurs after most nests have been initiated and, given the length of the nesting and brood rearing season, there is little potential for successful renesting.

The nest initiation window for least tern coincides more closely with the late‐spring rise, although the peak of initiation still precedes the mid‐June peak (Figure 8). The peak of least tern nest initiation also often occurs prior to the late‐spring rise, but the later overall nest initiation window and shorter nesting and brood rearing periods provide more potential for renesting following a late‐spring rise.

### 3.2 Emergent sandbar availability model

We found the median annual windows the species could have initiated a nest and successfully fledged chicks (success window) to be highest in the LPR reach and lowest in the historical AHR (Table 3). However, the median success window for piping plover was minimal in all reaches (<5 days). The success window for least terns was somewhat higher in the LPR and contemporary AHR reaches. However, the potential for season‐long successful nesting was <30% for both species in both reaches. Overall, the model predicted limited potential for successful fledging by either species in the historical AHR and piping plover in the contemporary reaches. The potential for successful fledging of least tern chicks was somewhat higher in the contemporary reaches, although the median window was only 3 weeks in the LPR and 2 weeks in the contemporary AHR. Overall, the potential for reproductive success was greatest in sequences of years with declining peak discharge magnitudes.

**Table 3:** Emergent sandbar habitat model output by reach including the median number of days each species could nest successfully (initiate a nest and fledge a chick) each year and the percent of years when no period existed when successful nesting could occur as well as the percent of years when the entire nesting season was suitable for successful nesting

Reach	Model Period	Median success window (days)		No success window (% of years)		Season‐long success window (% of years)	
		Piping plover	Least tern	Piping plover	Least tern	Piping plover	Least tern
LPR reach	1954–2012	4	21	42	17	22	25
Contemporary AHR	1954–2012	0	14	53	29	25	29
Historical AHR	1895–1938	0	0	84	68	5	7

### 3.3 Emergent sandbar availability model performance and sensitivity

Sandbar model performance in predicting the potential for nest inundation was assessed through examination of observed nest losses in relation to habitat forming and inundating flows. In 1947, a mean daily peak discharge of 394 cm occurred in the AHR on 23 June. On‐channel nests observed in 1948 were inundated twice even though the highest mean daily peak discharge during the 1948 nesting season was 127 cm which is well below the previous year peak of 394 cm. This indicates that sandbars used by the species in 1948 were formed to an elevation well below the stage associated with the previous year peak.

In 1978, discharge in the AHR peaked at 297 cm. Faanes (1983) reported all on‐channel least tern and piping plover nests in 1979 were inundated by flows of 85 cm. In 2014, two least tern nests were initiated within the AHR following the 2013 high flow event that had a peak mean daily discharge of 286 cm (Baasch, 2014); those nests were inundated at 82 cm. The contemporary AHR model predicted that the 1979 nests would have been inundated at 123 cm and 2014 nests inundated at 116 cm.

Similarly, a discharge of 2,379 cm within the LPR at Louisville in 2008 produced sandbar habitat inundated by a discharge of 595 cm in 2009, flooding 50 least tern and 14 piping plover nests (Brown & Jorgensen, 2009). In 2010, a mean daily peak discharge of 3,398 cm at Louisville produced sandbar habitat inundated in 2011 at a peak discharge of 940 cm flooding all least tern and piping plover nests observed on the river (Brown et al., 2011). The contemporary LPR model predicted that the 2009 nests would have been inundated at 968 cm and 2011 nests inundated at 1,489 cm.

As noted previously, other analyses have assumed sandbars build to the water surface during peak flow events (Parham, 2007; USFWS, 2006). If that assumption were accurate, we would not have expected to observe significant nest losses in any of the above cases. The emergent sandbar habitat model, which utilized sandbar heights of 0.45 m below peak stage in the AHR and 0.61 m in the LPR, still overpredicts the discharge necessary to inundate sandbars used by the species. Consequently, model sandbar heights of appear to be conservatively high, overestimating the potential for reproductive success. Conversely, previous models assuming sandbars build to the peak water surface seriously underestimate the potential for nest loss due to inundation and overestimate the potential for reproductive success.

The emergent sandbar model Monte Carlo sensitivity analysis indicates the median success window for all reaches was insensitive to stage–discharge and quite sensitive to sandbar height input variables. In all cases, over 90% of the variance in success window was attributable to sandbar height (Table 4). Our sensitivity analysis indicates that sandbar height assumption has a much larger influence on model results than the stage–discharge relationships used to characterize the channel at use locations. For example, increasing the LPR reach bar height from 0.61 m below peak stage to 0.00 m below peak stage reduced the percent of years with no potential for piping plover reproductive success from 42% of years to 5% of years. The percent of years with no potential for least tern reproductive success was reduced from 17% of years to 0% of years.

**Table 4:** Emergent sandbar habitat model median success window sensitivity to stage–discharge and sandbar height input variable values. Monte Carlo sensitivity analysis utilized stage‐increase per unit discharge range from 70% to 130% of default model value. Sandbar height range for Associated Habitat Reach (AHR) reaches ranged from 0 to 0.91 m below formative stage. Sandbar height range for lower Platte River (LPR) Reach ranged from 0.15 to 1.07 m below formative stage

Reach	Stage–discharge (% of variance)		Sandbar height (% of variance)	
	Piping plover	Least tern	Piping plover	Least tern
LPR reach	6.0	6.1	94.0	93.9
Contemporary AHR	3.6	5.3	96.4	94.7
Historical AHR	2.0	3.9	98.0	96.1

## 4 Discussion

If, as hypothesized in Platte River literature, least tern and piping plover are physiologically adapted to begin nesting on the Platte River concurrent with the recession of the spring rise, we would expect this to be reflected in the timing of species nest initiation. This adaptation is apparent in analyses of least tern nesting on the lower Mississippi River where the annual hydrograph peaks in April and tern nest initiation period begins in May, following the peak (Dugger et al., 2002). In the CPR and LPR, both species begin initiating nests in May, before the late‐spring rise which typically occurs in mid‐June. The median nest initiation dates for piping plovers and least terns are 15 May and 10 June, respectively, which is prior to and concurrent with the late spring rise. Given a majority of nests are initiated by these species prior to the late‐spring rise, we cannot conclude they are currently physiologically adapted to the hydrology of the Platte River. One could argue these species were historically adapted to the hydrology of the Platte River, and contemporary nest initiation periods have been influenced by habitat modification or climate change. However, the timing of the late spring rise has not changed. Consequently, these species would have historically had to begin initiating nests much earlier or much later. There is no evidence to suggest these species historically initiated a preponderance of nests in March and April or began initiating nests in late June or July.

Regardless of any physiological adaptation, a decline of on‐channel least tern and piping plover use and productivity in the AHR has been inferred from the reduction in AHR channel width from the predevelopment period, a reduction in the magnitude of the spring rise resulting in unsuitably low sandbar habitat likely to be inundated during the nesting season, a lack of on‐channel nesting in the contemporary AHR, and species use of the contemporary LPR (USFWS, 2006). This inference assumes that (1) the LPR is a functional analog for the historical AHR and (2) the contemporary LPR (and by extension the historical AHR) supports reproductive levels sufficient to maintain species subpopulations.

The assumption that the LPR is a functional analog for the historical AHR can be evaluated through comparisons of hydrology, channel form, and the potential for successful species nesting. The mean annual hydrograph of the LPR and historical AHR is similar in that there are pronounced early and late spring rises with the late spring rise occurring in mid‐June. However, the historical AHR channel was much wider than the contemporary LPR and flows were approximately 50% lower (Figures 5 and 6). Consequently, stage increase in the historical AHR during the late spring rise and the associated ability to build suitably high sandbars was likely more limited than the contemporary LPR. These differences are apparent in the divergent sandbar model results for the two reaches (Table 3) and do not support the assumption that the contemporary LPR is a functional analog of the historical AHR.

It was also assumed that the contemporary LPR (and by extension the historical AHR) channel supports least tern and piping plover reproductive levels that are sufficient to maintain species subpopulations. Within the contemporary AHR and LPR, piping plovers nest from late April to early August with the highest proportion of nests being initiated during May. Least terns breed and nest from mid‐May to early August with the highest nesting incidence occurring in early June. As a result, a preponderance of nests is often initiated prior to the late spring rise and is lost to inundation. The potential for successful reproduction is then dependent upon renesting. The timing of the late spring rise in relation to the piping plover nesting season severely limits the potential for successful reproduction as chicks from nests initiated in late June or early July would not fledge until September. Least terns have a greater potential for successful renesting given their incubation and brood rearing period is about 2 weeks shorter than piping plovers.

Three‐year running average fledge ratios of 1.13 fledglings/pair for piping plovers and 0.70 fledglings/pair for least terns have been proposed as necessary to maintain a stable to growing piping plover and least tern populations in the AHR (Lutey, 2002). The historical AHR model results indicate some potential for piping plover reproductive success in 16% of years and least tern success in 32% of years. Accordingly, piping plovers would have needed to average 7.06 fledglings/pair during those 16% of years in order to support an average fledge ratio 1.13 fledglings/year. This is not possible unless all breeding pairs successfully fledged two broods per year. Least terns would have needed to produce 2.19 fledglings/pair during the 32% of years that a potential for reproductive success existed to average 0.70 fledglings/year. Least tern fledge ratios exceeding 2.0 fledglings/pair have not been observed on the Platte River even in the absence of flooding.

The potential for maintenance of stable on‐channel piping plover subpopulations in the contemporary AHR and LPR segments is also low. During years that have a potential for reproductive success, average piping plover fledge ratios required to maintain a stable subpopulation within the contemporary AHR (1.95 fledglings/pair) and LPR (2.40 fledglings/pair) are substantially higher than average fledge ratios observed on constructed habitats within the AHR, 2010–2015 (Cahis & Baasch, 2016). Maintenance of a stable least tern subpopulation in the contemporary AHR would require a fledge ratio of 0.99 fledglings/pair and LPR would require a fledge ratio of 0.84 fledglings/breeding pair during years when a potential for successful nesting occurred. While we have consistently observed fledge ratios in this range on off‐channel habitats within the AHR, similar fledge ratios are uncharacteristic for in‐channel sandbar habitats on the AHR or LPR.

Why then, do these species occur along the Platte River? An alternative view is suggested by historical and contemporary species use of both in‐ and off‐channel habitats. The earliest species observations in the AHR include documentation of nesting on natural sandbars, artificially created on‐channel islands comprised of spoil from a sandpit operation, and at an off‐channel sandpit (Wycoff, 1960). In the lower portion of the basin, records in the late 1800s include off‐channel nesting at rainwater basins and along lake shorelines (Ducey, 2000; Pitts, 1988).

In the contemporary LPR and AHR, these species routinely make use of off‐channel habitats regardless of whether on‐channel habitat is available or not (Baasch, 2014; Brown & Jorgensen, 2008, 2009, 2010; Brown et al., 2011, 2012, 2013). These off‐channel habitats have been viewed as an inferior alternative to on‐channel nesting habitat that became necessary as on‐channel habitat suitability declined over historical timeframes (National Research Council, 2005; Sidle & Kirsch, 1993). However, given the limited potential for consistent success of on‐channel nesting in the CPR and LPR and perennial use of off‐channel habitat, these alternative habitats may have actually allowed the species to expand into and persist in a basin where the hydrology is not ideally suited to their reproductive ecology.

Since 2007, the program has implemented an Adaptive Management Plan to explore key uncertainties related to the response of least tern and piping plover to management actions on the CPR (PRRIP, 2006). A primary question is the role of on‐ and off‐channel least tern and piping plover nesting habitat. The results of substantial investments in on‐ and off‐channel mechanical habitat creation, flow and species monitoring, and related data analysis and synthesis have led the Program to re‐examine the benefits of management strategies that place a heavy emphasis on on‐channel habitat. The program has shifted toward species management activities focused primarily on maintaining a substantial supply of suitable off‐channel habitat while providing a limited amount of on‐channel habitat. This shift in management for least tern and piping plover based on program learning represents a successful application of adaptive management, unique among riverine restoration programs attempting adaptive management at large scales.

## Conflict of Interest

The authors are unaware of any real or perceived conflicts of interest.
